# Supplementary material for: Clinical and psychosocial factors associated with domestic violence among men and women in Kandy, Sri Lanka
Source: PLOS Glob Public Health. 2022 Apr 1;2(4):e0000129. doi: 10.1371/journal.pgph.0000129 (PMC10021245; doi:10.1371/journal.pgph.0000129)
Supplement: S1 Table — (DOCX) [file pgph.0000129.s001.docx]

**S1 Table. Humiliation, Afraid, Rape, Kick (HARK) questionnaire (English translated version).**

| *Within the last year:* | Yes | No | Refused |
| --- | --- | --- | --- |
| Have you been afraid of your partner or another family member who lives in your household? | 1 | 0 | 88 |
| Have you been kicked, hit, slapped or otherwise physically hurt by your partner or another family member who lives in your household? | 1 | 0 | 88 |
| Have you been raped or forced to have any kind of forced (i.e. against your will) sexual activity by your partner or another family member who lives in your household? | 1 | 0 | 88 |
| Have you been humiliated or emotionally abused in other ways by your partner or another family member who lives in your household? | 1 | 0 | 88 |
