# Supplementary material for: Clinical and psychosocial factors associated with domestic violence among men and women in Kandy, Sri Lanka
Source: PLOS Glob Public Health. 2022 Apr 1;2(4):e0000129. doi: 10.1371/journal.pgph.0000129 (PMC10021245; doi:10.1371/journal.pgph.0000129)
Supplement: S2 Table — (DOCX) [file pgph.0000129.s002.docx]

**S2 Table. Social support questions derived from a social capital community survey in the North Central Province of Sri Lanka.**

| *In general, do you agree or disagree with the following statements:* | Agree strongly | Agree somewhat | Neutral  (n, %)* | Disagree somewhat | Disagree strongly |
| --- | --- | --- | --- | --- | --- |
| I have a person in this household with whom I can share my joy and grief | 0 | 1 | 2  (5, 0.6) | 3 | 4 |
| People in this household are willing to help in difficult situations | 0 | 1 | 2  (3, 0.4) | 3 | 4 |
| I have friends or relatives in this neighbourhood with whom I can share my joy and grief | 0 | 1 | 2  (30, 3.6) | 3 | 4 |
| I feel at home in this neighbourhood | 0 | 1 | 2  (21, 2.5) | 3 | 4 |

*Neutral responses were combined with ‘agree strongly’ and ‘agree somewhat’ responses; n = number of neutral responses to statement; % = proportion of responses to the statement that were neutral.
