# Supplementary material for: Clinical and psychosocial factors associated with domestic violence among men and women in Kandy, Sri Lanka
Source: PLOS Glob Public Health. 2022 Apr 1;2(4):e0000129. doi: 10.1371/journal.pgph.0000129 (PMC10021245; doi:10.1371/journal.pgph.0000129)
Supplement: S3 Table — (DOCX) [file pgph.0000129.s003.docx]

**S3 Table. Sociodemographic characteristics by any exposure to past-year domestic violence (DV)**

|  | **Overall (N=846)** | | **Females (n=488)** | | **Males (n=358)** | |
| --- | --- | --- | --- | --- | --- | --- |
|  | DV  N (%) | No DV  N (%) | DV  N (%) | No DV  N (%) | DV  N (%) | No DV  N (%) |
| **Sex** |  |  |  |  |  |  |
| Male | 64 (40.8) | 294 (42.7) |  |  |  |  |
| Female | 93 (59.2) | 395 (57.3) |  |  |  |  |
| **Age** |  |  |  |  |  |  |
| 18 to 30 | 100 (63.7) | 402 (58.4) | 67 (72.0) | 260 (65.8) | 33 (51.6) | 142 (48.3) |
| 31 to 45 | 35 (22.3) | 165 (24.0) | 16 (17.2) | 90 (22.8) | 19 (29.7) | 75 (25.5) |
| 46 to 90 | 22 (14.01) | 122 (17.7) | 10 (10.8) | 45 (11.4) | 12 (18.8) | 77 (26.2) |
| **Ethnicity** |  |  |  |  |  |  |
| Sinhala | 136 (86.6) | 606 (88.0) | 82 (88.2) | 360 (91.1) | 54 (84.4) | 246 (83.7) |
| Non-Sinhala | 21 (13.4) | 83 (12.1) | 11 (11.8) | 35 (8.9) | 10 (15.6) | 48 (16.3) |
| **Highest education level** |  |  |  |  |  |  |
| Passed A/L or completed tertiary | 70 (44.6) | 348 (50.5) | 45 (45.2) | 218 (55.2) | 28 (43.8) | 130 (44.2) |
| Passed O/L | 40 (25.5) | 186 (27.0) | 25 (26.9) | 100 (25.3) | 15 (23.4) | 86 (29.3) |
| Completed between grades 1-10, or no schooling | 47 (29.9) | 155 (22.5) | 26 (28.0) | 77 (19.5) | 21 (32.8) | 78 (26.5) |
