# Supplementary material for: Clinical and psychosocial factors associated with domestic violence among men and women in Kandy, Sri Lanka
Source: PLOS Glob Public Health. 2022 Apr 1;2(4):e0000129. doi: 10.1371/journal.pgph.0000129 (PMC10021245; doi:10.1371/journal.pgph.0000129)
Supplement: S4 Table — (DOCX) [file pgph.0000129.s004.docx]

**S4 Table. Clinical and psychosocial factors associated with domestic violence (DV) in Kandy, Sri Lanka, stratified by type of abuse.***

|  | Physical/sexual abuse (n=26) | | Psychological abuse only (n=128) | |
| --- | --- | --- | --- | --- |
|  | **Model 1** | **Model 2** | **Model 1** | **Model 2** |
|  | **OR (95% CI)** | **OR (95% CI)** | **OR (95% CI)** | **OR (95% CI)** |
| *Clinical factors* |  |  |  |  |
| Depression symptoms (PHQ-9≥10) |  |  |  |  |
| No | 1.00 | 1.00 | 1.00 | 1.00 |
| Yes | 4.94 (2.22-10.97) | 4.69 (2.09-10.51) | 2.94 (1.83-4.71) | 2.81 (1.75-4.53) |
| Any suicidal ideation (PHQ item 9) |  |  |  |  |
| No | 1.00 | 1.00 | 1.00 | 1.00 |
| Yes | 9.36 (3.91-22.41) | 8.82 (3.65-21.34) | 5.53 (3.15-9.73) | 5.24 (2.96-9.26) |
| Ever diagnosed with mental illness |  |  |  |  |
| No | 1.00 | 1.00 | 1.00 | 1.00 |
| Yes | 4.12 (0.87-19.48) | 4.05 (0.85-19.28) | 3.63 (1.54-8.59) | 3.71 (1.56-8.81) |
| Previously self-harmed |  |  |  |  |
| No | 1.00 | 1.00 | 1.00 | 1.00 |
| Yes | 14.24 (5.47-37.06) | 13.35 (5.05-35.31) | 5.6 (2.75-11.42) | 5.3 (2.57-10.90) |
| Harmful alcohol use (AUDIT≥8) |  |  |  |  |
| No | 1.00 | 1.00 | 1.00 | 1.00 |
| Yes | 0.59 (0.14-2.57) | 0.55 (0.13-2.38) | 1.15 (0.65-2.01) | 1.08 (0.61-1.91) |
| Chronic illness/disability |  |  |  |  |
| No | 1.00 | 1.00 | 1.00 | 1.00 |
| Yes | 1.52 (0.56-4.13) | 1.57 (0.58-4.26) | 1.68 (1.02-2.75) | 1.68 (1.02-2.76) |
| *Social support factors* |  |  |  |  |
| Household member to share joy and grief |  |  |  |  |
| Yes | 1.00 | 1.00 | 1.00 | 1.00 |
| No | 8.93 (2.62-30.42) | 7.82 (2.25-27.10) | 10.2 (4.79-21.74) | 9.94 (4.65-21.26) |
| Household member supportive in difficult situations |  |  |  |  |
| Yes | 1.00 | 1.00 | 1.00 | 1.00 |
| No | 6.97 (1.35-35.84) | 5.77 (1.10-30.31) | 15.61 (6.51-37.44) | 14.76 (6.12-35.58) |
| Community member to share joy and grief |  |  |  |  |
| Yes | 1.00 | 1.00 | 1.00 | 1.00 |
| No | 2.71 (1.14-6.4) | 2.71 (1.14-6.46) | 2.42 (1.49-3.93) | 2.43 (1.49-3.96) |
| Feel at home in community |  |  |  |  |
| Yes | 1.00 | 1.00 | 1.00 | 1.00 |
| No | 3.26 (1.26-8.47) | 3.34 (1.27-8.79) | 2.82 (1.65-4.84) | 2.96 (1.72-5.10) |
| *Household composition* |  |  |  |  |
| Civil status |  |  |  |  |
| Married | 1.00 | 1.00 | 1.00 | 1.00 |
| Single | 0.35 (0.15-0.83) | 0.4 (0.16-0.96) | 0.92 (0.55-1.53) | 1.02 (0.61-1.71) |
| Divorced | 2.12 (0.23-19.3) | 2.27 (0.25-20.97) | 3.03 (1.14-8.04) | 2.95 (1.11-7.86) |
| Number of children |  |  |  |  |
| None | 1.00 | 1.00 | 1.00 | 1.00 |
| One to two | 2.13 (0.83-5.47) | 1.78 (0.67-4.75) | 1.34 (0.77-2.31) | 1.20 (0.68-2.11) |
| Three or more | 3.22 (0.66-15.87) | 2.38 (0.46-12.42) | 1.92 (0.89-4.12) | 1.61 (0.72-3.6) |
| Nuclear family |  |  |  |  |
| No | 1.00 | 1.00 | 1.00 | 1.00 |
| Yes | 1.04 (0.49-2.20) | 1.11 (0.52-2.38) | 0.74 (0.51-1.08) | 0.76 (0.52-1.11) |
| Presence of in-laws |  |  |  |  |
| No | 1.00 | 1.00 | 1.00 | 1.00 |
| Yes | 2.23 (0.80-6.19) | 2.00 (0.71-5.61) | 2.16 (1.29-3.60) | 2.12 (1.27-3.54) |
| Extended family (biological) |  |  |  |  |
| No | 1.00 | 1.00 | 1.00 | 1.00 |
| Parent/grandparent/grandchild | 1.38 (0.40-4.84) | 1.35 (0.38-4.74) | 0.49 (0.17-1.41) | 0.48 (0.17-1.37) |
| *Other study factors* |  |  |  |  |
| Sex |  |  |  |  |
| Male | 1.00 | 1.00 | 1.00 | 1.00 |
| Female | 1.41 (0.65-3.09) |  | 1.02 (0.70-1.49) |  |
| Age |  |  |  |  |
| 18 to 30 | 1.00 | 1.00 | 1.00 | 1.00 |
| 31 to 45 | 0.44 (0.15-1.31) |  | 0.97 (0.62-1.52) |  |
| 46 to 90 | 0.45 (0.13-1.53) |  | 0.80 (0.47-1.38) |  |
| Ethnicity |  |  |  |  |
| Sinhala | 1.00 | 1.00 | 1.00 | 1.00 |
| Non-Sinhala | 0.26 (0.04-1.94) |  | 1.35 (0.80-2.30) |  |
| Highest education level |  |  |  |  |
| Passed A/L or completed tertiary | 1.00 | 1.00 | 1.00 | 1.00 |
| Passed O/L | 1.40 (0.58-3.39) |  | 1.00 (0.62-1.60) |  |
| Completed between grades 1-10, or no schooling | 1.50 (0.60-3.73) |  | 1.51 (0.97-2.36) |  |

OR = Odds ratio; CI = Confidence Interval. *To avoid statistical disclosure, counts and proportions are not shown.
Model 1: Clinical factors adjusted for age; household and social support factors adjusted for age and ethnicity. Other study factors are unadjusted.
Model 2: Additionally adjusting for educational attainment.
