# Supplementary material for: Clinical and psychosocial factors associated with domestic violence among men and women in Kandy, Sri Lanka
Source: PLOS Glob Public Health. 2022 Apr 1;2(4):e0000129. doi: 10.1371/journal.pgph.0000129 (PMC10021245; doi:10.1371/journal.pgph.0000129)
Supplement: S5 Table — (DOCX) [file pgph.0000129.s005.docx]

**S5 Table. Clinical and psychosocial factors associated with domestic violence (DV) – sensitivity analysis restricted to household-based participants in Kandy, Sri Lanka (N=475).**

|  | DV (n=84) | No DV (n=391) | Model 1 | Model 2 |
| --- | --- | --- | --- | --- |
|  | **N (%)** | **N (%)** | **OR (95% CI)** | **OR (95% CI)** |
| *Clinical factors* |  |  |  |  |
| Depression symptoms (PHQ-9≥10) |  |  |  |  |
| No | 60 (71.4) | 357 (91.3) | 1.00 | 1.00 |
| Yes | 24 (28.6) | 34 (8.7) | 4.13 (2.28-7.48) | 3.87 (2.12-7.08) |
| Any suicidal ideation (PHQ item 9) |  |  |  |  |
| No | 65 (77.4) | 370 (94.6) | 1.00 | 1.00 |
| Yes | 19 (22.6) | 21 (5.4) | 5.30 (2.68-10.45) | 4.95 (2.49-9.82) |
| Ever diagnosed with mental illness |  |  |  |  |
| No | 76 (90.5) | 381 (97.4) | 1.00 | 1.00 |
| Yes | 8 (9.5) | 10 (2.6) | 3.93 (1.50-10.32) | 4.02 (1.52-10.64) |
| Previously self-harmed |  |  |  |  |
| No | 69 (82.1) | 381 (97.4) | 1.00 | 1.00 |
| Yes | 15 (17.9) | 10 (2.6) | 8.05 (3.46-18.72) | 7.28 (3.08-17.24) |
| Harmful alcohol use (AUDIT≥8) |  |  |  |  |
| No | 72 (85.7) | 341 (87.2) | 1.00 | 1.00 |
| Yes | 12 (14.3) | 50 (12.8) | 1.22 (0.61-2.42) | 1.06 (0.52-2.15) |
| Chronic illness/disability |  |  |  |  |
| No | 67 (79.8) | 336 (85.9) | 1.00 | 1.00 |
| Yes | 17 (20.2) | 55 (14.1) | 1.63 (0.88-3.00) | 1.59 (0.86-2.95) |
| *Social support factors* |  |  |  |  |
| Household member to share joy and grief |  |  |  |  |
| Yes | 69 (82.1) | 385 (98.5) | 1.00 | 1.00 |
| No | 15 (17.9) | 6 (1.5) | 14.88 (5.46-40.52) | 14.06 (5.12-38.64) |
| Household member supportive in difficult situations |  |  |  |  |
| Yes | 68 (81.0) | 387 (99.0) | 1.00 | 1.00 |
| No | 16 (19.0) | * | 26.61 (8.40-84.28) | 25.56 (7.97-82.02) |
| Community member to share joy and grief |  |  |  |  |
| Yes | 63 (75.0) | 354 (90.5) | 1.00 | 1.00 |
| No | 21 (25.0) | 37 (9.5) | 3.09 (1.69-5.65) | 3.19 (1.73-5.87) |
| Feel at home in community |  |  |  |  |
| Yes | 65 (77.4) | 361 (92.3) | 1.00 | 1.00 |
| No | 19 (22.6) | 30 (7.7) | 3.42 (1.81-6.46) | 3.59 (1.88-6.84) |
| *Household composition* |  |  |  |  |
| Civil status |  |  |  |  |
| Married | 46 (54.8) | 219 (56.0) | 1.00 | 1.00 |
| Never married | 33 (39.3) | 161 (41.2) | 0.75 (0.41-1.36) | 0.82 (0.44-1.51) |
| Divorced, separated or widowed | 5 (6.0) | 11 (2.8) | 2.60 (0.81-8.37) | 2.55 (0.79-8.25) |
| Number of children |  |  |  |  |
| None | 36 (42.9) | 195 (49.9) | 1.00 | 1.00 |
| One to two | 37 (44.0) | 149 (38.1) | 2.13 (1.14-3.98) | 1.95 (1.03-3.69) |
| Three or more | 11 (13.1) | 47 (12.0) | 2.49 (0.96-6.46) | 2.1 (0.78-5.64) |
| Nuclear family |  |  |  |  |
| No | 48 (57.1) | 193 (49.4) | 1.00 | 1.00 |
| Yes | 36 (42.9) | 198 (50.6) | 0.71 (0.44-1.16) | 0.73 (0.45-1.19) |
| Presence of in-laws |  |  |  |  |
| No | 66 (78.6) | 345 (88.2) | 1.00 | 1.00 |
| Yes | 18 (21.4) | 46 (11.8) | 2.24 (1.2-4.15) | 2.14 (1.15-3.99) |
| Extended family (biological) |  |  |  |  |
| No | 79 (94.0) | 366 (93.6) | 1.00 | 1.00 |
| Parent/grandparent/grandchild | 5 (6.0) | 25 (6.4) | 0.95 (0.35-2.61) | 0.94 (0.34-2.59) |

OR = Odds ratio; CI = Confidence Interval. *To avoid statistical disclosure, low counts (<5) are not shown.
Model 1: Clinical factors adjusted for age; household and social support factors adjusted for age and ethnicity.
Model 2: Additionally adjusting for educational attainment.
