# Supplementary material for: Clinical and psychosocial factors associated with domestic violence among men and women in Kandy, Sri Lanka
Source: PLOS Glob Public Health. 2022 Apr 1;2(4):e0000129. doi: 10.1371/journal.pgph.0000129 (PMC10021245; doi:10.1371/journal.pgph.0000129)
Supplement: Attachment — Submitted filename: Response to Reviewers letter.docx [file pgph.0000129.s006.docx]

|  |  |  |
| --- | --- | --- |
|  | | |
|  | | **Translational Health Research Institute**  Building 3, Campbelltown Campus  Locked Bag 1797  Penrith NSW 2751 Australia  T +61 2 4620 3669 F +61 2 4620 3299 E [p.bandara@westernsydney.edu.au](mailto:p.bandara@westernsydney.edu.au) |
| Dr. Catherine Kyobutungi Editor-in-Chief, *PLOS Global Health*  Prof. Madhukar Pai Editor-in-Chief, *PLOS Global Health* |  |  |
| 26 November 2021 | | |

Dear Dr. Kyobutungi and Prof. Pai,

**RE: Re-submission of PGPH-D-21-00266 – Clinical and psychosocial factors associated with domestic violence among men and women in Kandy, Sri Lanka.**

We would firstly like to take this opportunity to thank the editors and reviewers for taking the time to review our paper. Please find enclosed a manuscript, revised according to editor and reviewer comments. Responses to specific comments are provided below. Changes to text are indicated, with additional text underlined.

**Journal Requirements:**

*1. Along with your ethics statement, please include a statement that formal consent was obtained (must state whether verbal/written) OR the reason consent was not obtained (e.g., anonymity).*

An additional note has been made in the Method (pg. 5, paragraph 2):

 All interviews were conducted by trained data collectors with a nursing or basic science degree in the participant’s preferred language (Sinhala, Tamil or English). The research was explained to each participant and formal written consent was obtained.

*2. Please include additional information regarding the survey or questionnaire used in the study and ensure that you have provided sufficient details that others could replicate the analyses. For instance, if you developed a questionnaire as part of this study and it is not under a copyright more restrictive than CC-BY, please include a copy, in both the original language and English, as Supporting Information.*

All study questionnaires and instruments used in this study in both English and original languages (Sinhala and Tamil) can be accessed from the University of Bristol data depository. We have added a reference in the methods.

All study questionnaires and instruments used in this study (in English and local languages) can be accessed from the University of Bristol data depository.[20]

20. Knipe D, Kidger J. Data from ACE & Self harm Sri Lanka (10-2020): University of Bristol; 2020. Available from: h<ttps://doi.org/10.5523/bris.37pg6mv6x35r12b98aoq4blcgs.>

*3. We noticed that you used “data not shown”/"unpublished data" in the manuscript. We do not allow these references, as the PLOS data access policy requires that all data be either published with the manuscript or made available in a publicly accessible database. Please either remove these references, or amend the supplementary material to include the referenced data.*

We have removed these references.

*4. Please update the completed 'Competing Interests' statement, including any COIs declared by your co-authors. If you have no competing interests to declare, please state "The authors have declared that no competing interests exist". Otherwise please declare all competing interests beginning with the statement "I have read the journal's policy and the authors of this manuscript have the following competing interests:"*

We have added a Competing Interests statement (pg. 21) acknowledging “the authors have declared that no competing interests exist.”

*5. Since your data is not available for proprietary reasons, please explain via email why the data is not available. Please also include the contact information for the third party organization that should be contacted should other researchers want to request access to this data and please include the full citation of where the data can be found. We also request that you verify with us via email that any researcher will be able to obtain the data set in the same manner that the you have obtained it. If you feel you are unwilling or unable to adhere to this policy, please explain your reasons by return email and your exemption request will be escalated to the editor for approval. Your exemption request will be handled independently and will not hold up the peer review process, but will need to be resolved should your manuscript be accepted for publication. One of the Editorial team will be in touch if they require more information.*

Data are available at the University of Bristol data repository, [data.bris](https://data.bris.ac.uk/data/):

Citation: Dee Knipe, Judi Kidger (2020): Data from ACE & Self harm Sri Lanka (10-2020). <https://doi.org/10.5523/bris.37pg6mv6x35r12b98aoq4blcgs>. Correspondence to Dr Duleeka W Knipe; [dee.knipe@bristol.ac.uk](mailto:dee.knipe@bristol.ac.uk).

Given the high sensitivity of the data, only researchers at verifiable institutions will be able to access data. Any requests will be reviewed by the University of Bristol Access Committee, which includes senior researchers and representatives from the University. Data will only be released once a controlled data access agreement has been signed by a nominated institutional signatory.

**Reviewers' comments:

Reviewer #1:**

*The paper analyses the drivers of Domestic violence for men and women in Sri Lanka. The research question is interesting and extremely relevant for public health discourse globally. However, the authors may consider a few changes before publishing this manuscript--*

A) *the third objective i.e. to explore how the associations may differ by sex and by type of abuse will be better achieved if the model is run on a combined sample of men and women in used and sex is interacted with the key drivers of DV.*
B) *The model may be repeated for types of abuse to observe if the associations are different.*

We thank the Reviewer for their comments. Sex was specified as a potential effect modifier *a priori*, therefore, models were stratified and tests of interaction conducted to examine differences. Likelihood ratio tests were conducted to compare model fit (p-values presented in Tables 3 and 4) for models with, or without (but adjusting for sex), an interaction term between sex and a given co-variate. There was no statistical evidence that the addition of the interaction terms improved the fitness of the models. The small frequencies in categories of type of abuse precluded statistical tests for interaction. This has been clarified in the revised manuscript (pg. 7, paragraph 2):

The association between clinical factors (depression symptoms, suicidal ideation, alcohol misuse, prior psychiatric disorder diagnosis, previous self-harm, chronic illness/disability) and psychosocial factors (perceived social support, household composition – civil status, number of children, family structure) and any DV were assessed overall using a series of adjusted logistic regression models, then stratified by sex. To examine effect modification by sex for other co-variates, likelihood ratio tests were conducted to compare model fit for models with, or without, an interaction term between sex and a given co-variate.

C) *A discussion on the associations of DV and other control indicators (age, education) may be added as there are limited resource on DV on men.*

We thank the Reviewer for their comment. As this was not specified as part of our pre-registered analysis plan and does not align with the key objectives of the study, we did not examine individual associations between potential confounders (e.g., age, ethnicity, education) and the outcome. However, we have presented the distribution of these factors in a supplementary descriptive table (S3 Table), with an additional note in the results.

Results (pg. 10, paragraph 1):

Sociodemographic factors identified *a priori* as potential confounders (age, ethnicity, educational attainment) were evenly distributed between DV and non-DV cases (S3 Table).

D) *Visualization techniques should be explored for narrating the results.*

We thank the Reviewer for their comment. We have added figures to visualise the odds ratios, supplied below:

**Fig 1. Clinical and psychosocial factors associated with past-year domestic violence, Kandy, Sri Lanka.**
(A) Clinical correlates of past-year domestic violence, adjusted for age. (B) Psychosocial correlates of past-year domestic violence, adjusted for age and ethnicity. The bold line indicates a null result.

**Fig 2. Clinical and psychosocial factors associated with past-year domestic violence by sex, Kandy, Sri Lanka.**
(A) Clinical correlates of past-year domestic violence, adjusted for age. (B) Psychosocial correlates of past-year domestic violence, adjusted for age and ethnicity. The bold line indicates a null result.

E) *There are minor grammatical errors (missing comma etc.).*

We thank the Reviewer for their comment. We have resolved grammatical errors in the revised manuscript.

F) *Overall it is a carefully designed and clearly written article and will make a good addition to the GPH.*

We thank the reviewer for their comment and time considering the manuscript.

**Reviewer #2:**

*Summary: the authors examined the association of clinical and psychosocial factors with domestic violence using a dataset from Kandy, Sri Lanka. I commend the authors for looking at this important issue in Sri Lanka and particularly examining the sex difference. However, I have concerns regarding their interpretation of the results and the conclusions they made based on their findings.*

We thank the Reviewer for their comments.

Major comments:

• Intro

1. *In general, the logic flow of the intro needs to be improved. Reading paragraph #2 makes me think this manuscript would mainly focus on men who experienced DV or at least sex difference. However, the manuscript was not written in this way.*

We thank the Reviewer for their comment. We have revised the Introduction for clarity.

2. *I don’t think the authors could provide prevalence of DV based on the data they used in this paper. It is a convenient sample and the sample size is very small. I would not call the calculated percentage of DV from this sample as prevalence of DV in Sri Lanka.*

We thank the Reviewer for their comment. We acknowledge that this estimate is potentially not generalizable to wider Sri Lanka. However, Kandy (where the data was sampled) was specified in our title. In addition, we have rephrased the language throughout the manuscript to indicate that the prevalence is reflective of the source population from which the data were sampled. Although the sample size is limited, given the scarce information available on men’s experience of DV we believe it is valuable to present a prevalence estimate. We extensively acknowledge the limitations of the sample in the Discussion.

• Study variables

3. *When did DV happen? The HARK study limits the timeframe of the violence variables as the past year. Should we think that this manuscript used the same method? This section needs to be revised to add more clarity and transparency.*

We thank the Reviewer for their comment. The timeframe was not changed. The HARK tool used for this study was supplied as supplementary material for transparency (also presented below). We have made an additional note on “past-year” abuse within the text (pg. 6, paragraph 1):

The HARK tool identifies four types of abuse – physical, sexual, humiliation/emotional abuse, and fear of an intimate partner in the past year. The questionnaire was broadened to include past-year abuse by any family member living in the household, not just by an intimate partner (S1 Table). The tool was then translated, back-translated and piloted in the two local languages (Sinhala and Tamil) with individuals in the outpatient department. No modifications were required after piloting with the local population. A HARK score of ≥1 indicates an experience of at least one form of past-year DV.

**S1 Table. Humiliation, Afraid, Rape, Kick (HARK) questionnaire (English translated version).**

| *Within the last year:* | Yes | No | Refused |
| --- | --- | --- | --- |
| Have you been afraid of your partner or another family member who lives in your household? | 1 | 0 | 88 |
| Have you been kicked, hit, slapped or otherwise physically hurt by your partner or another family member who lives in your household? | 1 | 0 | 88 |
| Have you been raped or forced to have any kind of forced (i.e. against your will) sexual activity by your partner or another family member who lives in your household? | 1 | 0 | 88 |
| Have you been humiliated or emotionally abused in other ways by your partner or another family member who lives in your household? | 1 | 0 | 88 |

• Discussion

4. *Again, I don’t think this current study could provide prevalence of DV in Sri Lanka. The language needs to be revised.*

We thank the Reviewer for their comment. As indicated in our response to comment 2, we have rephrased the language to indicate that the prevalence estimates provided are reflective of the source population (Kandy) from which the data is sampled. We also noted the following in the limitations section of the Discussion (pg. 19, paragraph 2):

Given participants were recruited based on the age and sex distribution of self-poisoning cases, the sample is predominantly a younger adult population (18-30 years), with women on average younger than men. It is possible that the prevalence of DV in older women and younger men was under-enumerated, distorting the overall prevalence for males and females in Kandy. However, as previously discussed, estimates were similar to previous studies.

5. *“A key finding was that men and women experienced DV to a similar degree in Sri Lanka”. This is a very vague conclusion. What does similar mean here? The findings only suggested in the sample there is no significant difference between men and women in terms of the association between psychological factors and DV. This conclusion was made well beyond the findings.*
6. *Page 14, line 31: “notably, the prevalence of DV was similar for men and women.” I didn’t find the language in the results section.*

We thank the Reviewer for their comments, we note that a description of the similar prevalence for men and women was provided in the results section (pg. 10, paragraph 1):

Similar rates of any DV were found for women (19% 95% CI 15%–23%) and men (18% 95% CI 14%–22%). Examination of different forms of abuse showed similar prevalence for women and men for psychological abuse (women: 16% 95% CI 13% - 19%; men: 15% 95% CI 12% - 20%) and physical/sexual abuse (women: 5% 95% CI 3% - 7%; men: 3% 95% CI 2% - 6%).

A “similar degree” refers to the similar prevalence. However, we acknowledge the phrasing may be vague, therefore have revised the language for clarity (pg. 16, paragraph 2):

A key finding was that men and women experienced a similar prevalence of DV in Kandy ~~to a similar degree in Sri Lanka~~.

7. *Table 4, for the social support factors, we actually see big a difference between females and males despite statistical insignificance. Note that, p-values are all at boarder line and the small sample size could contribute to that. It would be inappropriate to conclude that there is no sex difference of the associations in Sri Lanka.*

We agree with the reviewer that the magnitude of the point estimates for social support factors differ between men and women. However, given the limitation in the sample size we were cautious to interpret these findings as evidence of a difference. We have rephrased the language in the Discussion to note differences in the point estimates and acknowledge the small sample size as a limitation in interpreting evidence of effect measure modification.

Discussion (pg. 16, opening paragraph):

There was no strong statistical evidence that associations differed by sex for almost all study variables, although there was some weak statistical evidence that lower social support in the household was associated with greater DV in women compared to men, and this was in the opposite direction for community support.

Discussion – strengths and limitations (pg. 19, paragraph 2):

Furthermore, due to low numbers in some categories (e.g. previously partnered individuals) and overall limitations in the sample size, statistical power was reduced, particularly for stratified analyses and interaction tests. It is possible the lack of statistical differences between men and women may be attributed to the small sample size, disguising actual population level differences in associations by sex.

8. *Page 15, line 43: the interpretation of the alcohol consumption finding is not clear.*

We thank the Reviewer for their comment. We have revised this interpretation for clarity (pg. 17, paragraph 2).

Although alcohol misuse has been linked with an increased risk of IPV victimisation among men and women in largely high-income countries,^2 3^ no association was found in the current study. ~~This was not surprising given a~~Alcohol consumption is not socially sanctioned in this context, especially for women,^4^ which may explain the low numbers reported and absence of an association.

9. *Page 15, line 50: the cited finding does not support the manuscript’s finding. The cited findings is that women with a disability had lower IPV rate than those without disability. However, the authors just found no statistical evidence between disability and DV among women. The cited finding is not the same as authors’ finding.*

We thank the Reviewer for their comment. The cited finding was used to highlight that the association between disability and IPV differs in Sri Lanka compared to international evidence. However, we agree with the Reviewer that the cited finding is not consistent with our finding. We have revised the language and acknowledge that further research is needed given the mixed evidence (pg. 17, paragraph 3).

~~While h~~Higher frequencies of chronic illness/disability have been reported among women experiencing IPV in other LMIC settings.^5-7^ Contrary to international evidence, according to Sri Lanka’s 2019 Women’s Wellbeing Survey, lower rates of past-year IPV have been reported among women with a disability versus no disability.^8^ There was no statistical evidence in the current study that chronic illness/disability was associated with DV among women. Given the mixed evidence, further studies are needed to explore how vulnerable populations such as those living with a disability may be affected.

Minor comments
*• Intro*

10. *Paragraph #2: what does “to a similar degree” mean specifically?*

We thank the Reviewer for their comment and acknowledge that this phrasing is vague. We have revised the wording to be more specific as follows (pg. 3, paragraph 2):

A WHO multi-country study showed that within Sri Lanka, men and women experienced a similar prevalence of past-year IPV ~~to a similar degree~~, signalling the importance of further DV research among men.

Overall, we thank the Editors and Reviewers for their time in considering this manuscript and for their comments.

Yours sincerely,

Ms. Piumee Bandara (Corresponding Author)

**References**

1. Bandara P, Knipe D, Munasinghe S, et al. Socioeconomic and geographic correlates of intimate partner violence in Sri Lanka: Analysis of the 2016 Demographic and Health Survey. *medRxiv* 2021 doi: <https://doi.org/10.1101/2021.03.21.21254059>

2. Ehrensaft MK, Moffitt TE, Caspi A. Is domestic violence followed by an increased risk of psychiatric disorders among women but not among men? A longitudinal cohort study. *American Journal of Psychiatry* 2006;163(5):885-92.

3. Devries KM, Child JC, Bacchus LJ, et al. Intimate partner violence victimization and alcohol consumption in women: A systematic review and meta‐analysis. *Addiction* 2014;109(3):379-91.

4. Hettige S, Paranagama D. Gender, alcohol and culture in Sri Lanka. *Alcohol, gender and drinking problems* 2005:167.

5. Ellsberg M, Jansen HA, Heise L, et al. Intimate partner violence and women's physical and mental health in the WHO multi-country study on women's health and domestic violence: an observational study. *The Lancet* 2008;371(9619):1165-72. doi: 10.1016/S0140-6736(08)60522-X

6. Pengpid S, Peltzer K. Lifetime spousal violence victimization and perpetration, physical illness, and health risk behaviours among women in India. *International journal of environmental research and public health* 2018;15(12):2737.

7. Campbell JC. Health consequences of intimate partner violence. *The lancet* 2002;359(9314):1331-36.

8. DCS. Women's Wellbeing Survey - 2019: Findings from Sri Lanka's first dedicated National Survey on Violence against Women and Girls. Sri Lanka: Department of Census and Statistics, Sri Lanka 2020.
